# Implant Stability Changes for Nonsubmerged and Submerged Protocols for a Single Implant Mandibular Overdenture Using Ball Attachment

**DOI:** 10.1155/2021/8269197

**Published:** 2021-09-16

**Authors:** Ahmed Salah, Karim Foda, Mohamed Farouk Abdalla, Marwa Abdel Aal, Amr Naguib, Nouran Abdel Nabi

**Affiliations:** Prosthodontics Department, Faculty of Dentistry, Cairo University, Giza, Egypt

## Abstract

**Objectives:**

To compare the changes in implant stability for the nonsubmerged (NS) and submerged (S) protocols for the single implant retained mandibular overdenture using ball attachment throughout a 24-month follow-up.

**Materials and Methods:**

Eighty completely edentulous patients were seeking to improve retention of their lower complete denture by installing a single implant in the midline of the completely edentulous mandible. At the day of implant installation, patients were randomized into 2 groups using sealed envelopes: the nonsubmerged (NS) and submerged (S) group. After a 3-month healing period, all patients were randomized using sealed envelopes into ball attachment and CM-LOC attachment. The Periotest readings (PTV) was recorded using the Periotest *M* device and was recorded every 3 months for the first year and then annually in the second year. The scope of this clinical trial focused only on results of the ball attachment. The Mann–Whitney *U* test was used for comparison between study groups for independent samples. Two-sided *p* values less than 0.05 was considered statistically significant.

**Results:**

There was no statistically significant difference in the mean change in PTV reading between the NS and S group at the different follow-up intervals. Initially, at the day of pickup (baseline) and 3-month follow-up, the mean PTV reading for the NS was greater than that of the S group (−4.471 ± 1.489, −4.391 ± 1.4727 (*p*=0.913)), while the S group has shown a greater improvement in PTV than the NS group after 6-month follow-up and continued throughout the 24-month follow-up (−5.730 ± 1.7804, −50855 ± 1.2581 (*p*=1)).

**Conclusion:**

Both the nonsubmerged and the submerged healing protocol have shown reliable Periotest readings using ball attachment for a single implant retained overdenture. The submerged group has resulted in a greater improvement in Periotest readings after the 12- and 24-month follow-up period when compared to the nonsubmerged group although this improvement was not statistically significant.

## 1. Introduction

Branemark first introduced the successful outcomes of the submerged surgical procedure in implant dentistry [1]. The submerged surgical protocol would enhance the process of new bone formation and remodeling by utilizing a two-stage surgical procedure, which first includes implant installation in the underlying bone and then a secondary-stage surgery after a period of osseointegartion [2]. The two-stage surgical protocol has proven to have good short- and long-term outcomes [3–5].

On the other hand, osseointegration has proven to be successfully achieved through a single-stage “nonsubmerged surgical protocol,” in which implants and the healing abutment are exposed in the oral cavity during the period of osseointegration [6, 7]. The nonsubmerged surgical protocol offers several advantages when compared to the submerged surgical protocol as it requires only a single-stage surgery which is more cost effective [8], it is more convenient to the patients reducing postoperative complications, and there is no microgap at the alveolar bone crest level [9]. But, the submerged surgical technique would be indicated in almost all cases, specifically in cases where bone augmentation is required, as it will decrease overloading of the implants during the osseointegartion period and would ensure optimum healing [10].

One of the principles of osseointegration is primary implant stability, which is essential for achieving short- and long-term success [11]. Primary implant stability is mainly associated with the mechanical engagement of the implant to the surrounding bone, whereas bone generation and remodeling phenomena determine the secondary (biological) stability [12, 13]. Bone quantity, bone quality, surgical technique, and implant design are factors that influence primary stability, while primary stability, bone remodeling, and implant surface conditions are considered as important factors that will influence secondary implant stability [14].

The Periotest device and Resonance Frequency Analysis (RFA) using the Osstell device have been considered as noninvasive methods to measure implant stability [15, 16]. Primary and secondary implant stability measurements using both devices have resulted in reproducible quantitative values. The Periotest is an electronic instrument designed to give quantitative measurements of the damping characteristics of the periodontal ligament surrounding a tooth, thus establishing a value for its mobility [17, 18]. The Periotest instrument comprises a hand piece containing a metal slug that is accelerated towards a tooth by an electromagnet. The contact duration of the slug on the tooth is measured by using an accelerometer. The software in the instrument is designed to relate contact time as a function of tooth mobility. The result is displayed digitally and audibly as Periotests values (PTVs) on a scale of −8 (low mobility) to 50 (high mobility). The technique has also been used to determine implant mobility, and typical values obtained were defined as ranging from −5 to 5, thus representing a narrower range over the scale of the instrument than for tooth mobility measurements. A stable implant will exhibit different stiffness characteristics compared with those of teeth that are connected by a periodontal ligament.

Implant supported over dentures has solved some of the problems of mandibular complete dentures. The MC Gill consensus 2002 and York consensus 2009 have stated that two implants installed in the mandible is considered to be the standard of care for completely edentulous patients [19–21]. Harder et al. and Cheng et al. have proved that a single implant installed in the midline can be an efficient treatment option as two implants installed in the mandible [22, 23]. Cordioli et al. introduced the idea of installing a single implant in the midline of a completely edentulous mandible to retain an overdenture [24]. The single implant retained mandibular overdenture is considered to be a cost-effective treatment option which has proved to have medium- to long-term survival rates [24–28].

The choice of the attachment system for the implant retained overdentures is considered to be of great importance as it has an impact on the overall patient satisfaction and the clinical success [29]. Ball and socket attachment has been the most popular unsplinted attachment used to retain a mandibular overdenture because of its simplicity and cost effectiveness [30]. Previous studies have reported that a single implant retained overdenture using ball or locator attachment to support an overdenture have proved satisfactory outcomes [24, 31–34].

The aim of this randomized clinical trial was to compare the changes in implant stability using the Periotest device for the nonsubmerged and submerged protocols for the single implant retained mandibular overdenture using ball attachment for a 24-month follow-up.

## 2. Materials and Methods

The study proposal was approved by the Ethical Committee of the Faculty of Dentistry, Cairo University, on June 13, 2016 (ethical approval No. 16/6/10) and is registered at http://www.pactr.org/(trial PACTR201803003085193). The guidelines of the World Medical Association were implemented in this clinical trial.

Eighty completely edentulous patients were recruited following strict inclusion criteria. All patients received a single implant in the midline of the edentulous mandible. At the day of implant installation, patients were randomized using sealed envelopes into two groups, nonsubmerged (NS) and submerged (S), and a 3-month healing period was allowed for all patients in both groups. The present study has followed the same inclusion criteria, sample size calculation, and all of the clinical relevant procedures of the trail carried out by Aal et al. [35]. All included patients (age ranging from 50–69 years) were recruited following strict inclusion criteria: glycosylated hemoglobin level ≥ 8, patients seeking to install a single median implant in the mandible, and for whom new dentures will be constructed were included. Patients with any condition that would contraindicate implant placement were excluded.

Patients were instructed to take a dose of 2 g of amoxicillin 2 hours before surgery. Local anesthesia was given in the lower anterior area; then, a small crestal incision was made in the area of implant installation, guided by the radiographic stent, which was converted to a surgical stent at the day of surgery. The surgical stent had a small opening in the area corresponding to the central incisors to help in implant installation. All implants installed in this study were ZDI implants with a tapered screw vent (Zimmer Dental, Warsaw, Ind), with a diameter of 3.7 mm and length of 10 mm, and SBM surface treatment which is a Soluble Blast Media of Hydroxyapatite (HA) crystals to create a rough texture, and then, the surface is cleaned with 20% acid solution. The description of the implant surface is fundamental, as it influences osseointegration and the health of the soft tissues [36, 37].

Drilling was carried out using the Zimmer Dental kit following the manufacturer's instructions. Following implant installation, all patients were instructed to be on Ibuprofen 400–600 mg every 6–8 hours; in addition to that, ice pack or cold compress was given to the patient to use for 2 hours postoperative implant installation.

This clinical trial followed up the changes in Periotest readings (PTV) for the nonsubmerged (NS) and submerged (S) groups after the 3 month of healing period (day of pickup) after which second randomization was followed.

### 2.1. Patient Distribution after 3-Month Healing Period (Day of Pickup)

**Figure 1:**
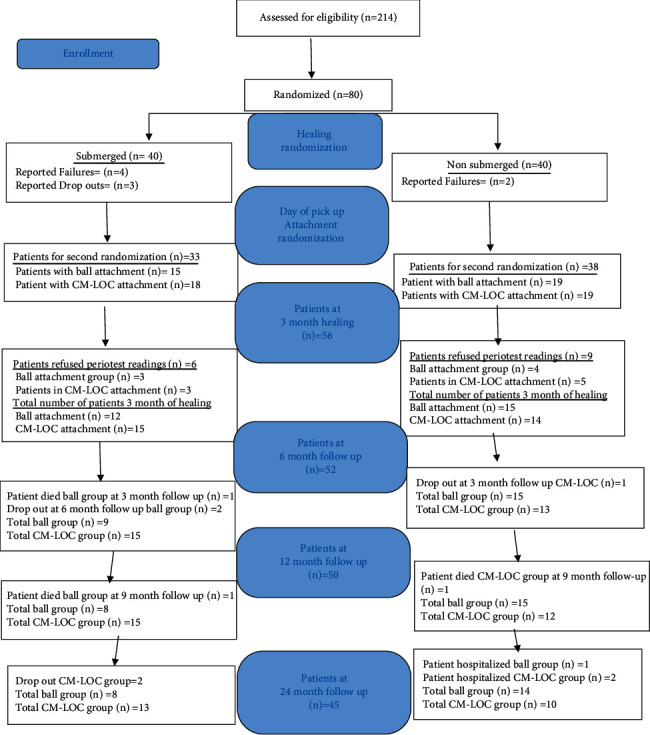
Consort flow diagram.

During the 3-month healing period, 4 patients have reported failure and 3 patients were counted as dropouts from the submerged group (S). While for the nonsubmerged group 2 patients have reported failure (Figure 1). The number of patients that were recalled after the 3-month healing period was 71: 33 patients in the submerged group (S) and 38 patients in the nonsubmerged group (NS). Failures in the submerged group were probably due to patients having bad oral hygiene during the healing period, while in the nonsubmerged group, they were mainly due to patients not following the instruction to eat soft food during the first few weeks subjecting the implant to great micromovements.

After a randomization process, the patients were divided in 2 groups (submerged vs. nonsubmerged protocol), and after 3 months of healing, they were randomly divided again into 2 groups (ball attachment vs. Cendres and Metaux attachment). In this study, we valued only the cases with ball attachment. The distribution of patients in each of the groups is described in Table 1. Randomization and allocation concealment were carried out by Amr Naguib, as he was responsible for preparing the envelopes used in randomization.

## 3. Intervention

### 3.1. Pickup of the Attachments

At the day of pickup, the healing abutments in the NS group were unscrewed and the ball (Zimmer dental implants) attachment was screwed in place with a torque of 30 N/cm, while for the S group, a small crestal incision was made at the area corresponding to the attachment.

**Figure 2:**
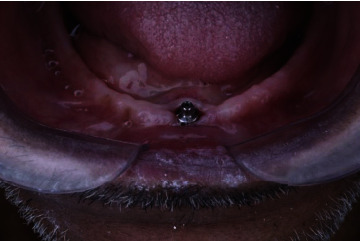
Ball attachment (Zimmer Company).

The ball attachment (Zimmer dental implants) compromises a male abutment with a gingival cuff height of 2 mm and 4 mm. The abutment was screwed onto the underlying implant using a specific screwdriver. The housings are made of titanium with a nylon transparent retentive insert that was supplied from Zimmer Company with the ball abutment (Figure 2).

The height of each attachment used in the following study was not standardized as it depends on the amount of mucosa present after healing which was different for each patient. The amount of keratinized mucosa was measured 3 month after healing, and two different ball attachments were used, 2 mm and 4 mm, accordingly.

**Figure 3:**
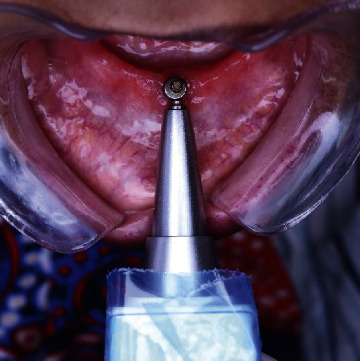
Measurement of Periotest values (PTV) of attachment using Periotest M.

After the attachments were screwed in place and the patients were instructed to sit in an upright position and not to move, the Periotest M (Medizintechnix Gulden e.K., Modautal, Germany) was directed to the midbuccal surface perpendicular to the long axis of the screwed attachment, and the tapping rod was directed at the bottom of an attachment as described by the manufacturer, to measure the implant stability (Periotest reading, damping effect = PTV) at the day of pickup (3 months after healing) which is considered to be the baseline reading. Five readings were recorded for each patient, and then, average reading was recorded in the patient file. This reading was considered to be the baseline reading (Figure 3). Ahmed Salah and Karim Foda were responsible for recording the PTV readings for all groups of patients throughout the 24-month follow-up period.

**Figure 4:**
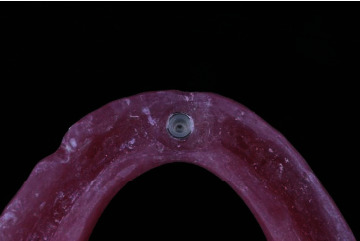
The nylon cap of ball attachment picked up in the fitting surface of the denture.

The ball attachment was screwed to the implant with a torque of 30 N/cm, with the corresponding matrix on top of it, Nylon matrix. The mandibular denture was then modified by cutting a small hole in the area corresponding to the attachment, and a red die was placed on top of the matrix to ensure that there was no interference between the matrix and the fitting surface of the modified denture. The mandibular denture was then checked for proper seating, and the occlusion with the maxillary denture was properly checked. All undercuts were blocked out in both attachments before pickup. The denture was then properly seated in place then a soft mix of Luxa pickup material (DMG) and then added to the hole of the modified denture; the patient was then asked to close in centric occlusion. After complete setting of Luxa pickup, the denture was removed and the pickup of the matrix was checked (Figure 4). All excess Luxa pick material was removed and then polished. Patients were recalled 3 days after pickup to check if there were any premature contacts or areas that required relief. This procedure was carried out for both attachments used in this study.

The Periotest reading (PTV) was recorded for all patients following all instructions that were given at the baseline reading (3 months after healing), five readings were recorded, and then, average was recorded in the patients' file. PTV was recorded every 3 months for the first 12 months and then annually at 24-month follow-up.

After the 3-month healing period, 33 patients were present in the S group and 38 patients in the NS group; 6 patients in the S group refused to have the Periotest readings, and 9 patients in the NS group resulting in a total of 56 patients after the 3-month healing period (baseline). At 3-month follow-up, 1 patient belonging to the ball attachment in the S group died and 1 patient was dropped out in the CM-LOC NS group, resulting in a total of 54 patients at 3-month follow-up. At 6-month follow-up, 2 patients belonging to ball attachment group in the S group were dropped out (1 male and 1 female) resulting in a total of 52 patients at 6-month follow-up. At 9-month follow-up, 1 patient from the ball attachment group in the S group died (male) and 1 patient from CM-LOC attachment in the NS group died (male), thus resulting in a total of 50 patients at 9-month follow-up. At 12-month follow-up, no dropouts occurred, while at 24-month follow-up, 2 patients belonging to the CM-LOC group in the S group were dropped outs (males), 1 patient in ball attachment group in NS group was hospitalized (female), and 2 patients from CM-LOC group NS were hospitalized (males), thus resulting in 45 patients at 24-month follow-up (Figure 1, Table 1).

Data were statistically described in terms of mean ± standard deviation (±SD). Comparison of numerical variables between the study groups was done using the Mann–Whitney *U* test for independent samples. Two-sided *p* values less than 0.05 were considered statistically significant. All statistical calculations were carried out using computer program IBM SPSS (Statistical Package for the Social Science; IBM Corp, Armonk, NY, USA) release 22 for Microsoft Windows.

## 4. Results

The interobserver consistency for the two readings of the PTV that was recorded by AS and KF for both groups, the NS and the S, during the different follow-up intervals using Cronbach`s alpha Statistics (Table 2). Results of the interobserver consistency showed a strong agreement for both groups at the different follow-up period, as the values at the different follow-up periods for both groups were greater than 0.7.

### 4.1. Comparing Mean Periotest Readings (PTV) between the Nonsubmerged (NS) and the Submerged (S) Group for the Ball Attachment Group at Different Follow-Up Intervals

**Figure 5:**
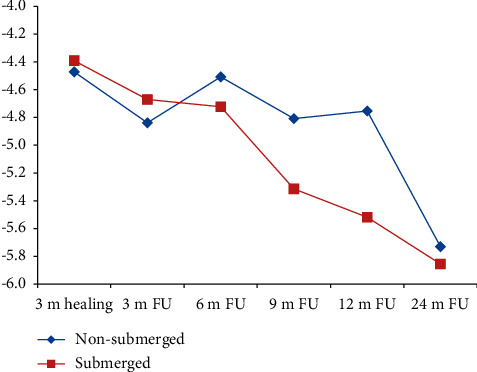
Mean PTV between NS and S in the ball attachment group.

There was no statically significant difference between the mean PTV readings of the NS and S groups for the ball attachment throughout the 24-month follow-up. At the day of pickup, which is considered to be baseline value, the NS group has recorded higher mean PTV readings than the S group (−4.471 ± 1.489, −4.391 ± 1.4727 (*p*=0.913)) (Table 3 and Figure 5). At 3-month follow-up, the mean PTV readings for the NS group were still higher than those for the S group. Then, starting from 6-month follow-up, the mean PTV readings for the S group was higher than those for the NS group throughout the 9-, 12-, and 24-month follow-up (−5.730 ± 1.7804, −50855 ± 1.2581 (*p*=0.1)) (Table 3 and Figure 5).

### 4.2. Changes in Mean Periotest Readings (PTV) between the Nonsubmerged (NS) and the Submerged (S) Group for the Ball Attachment Group at Different Follow-Up Intervals

**Figure 6:**
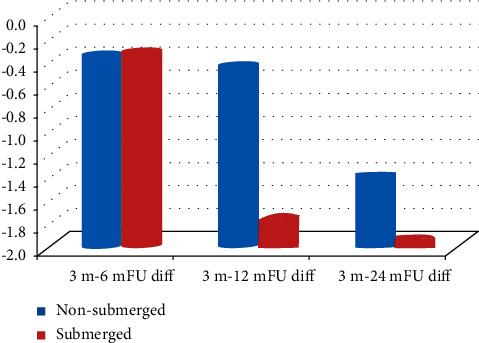
Mean PTV difference between NS and S in the ball attachment group.

When having a closer look at the changes of mean PTV readings for the ball attachment in the first 6 months, 12 months, and 24 months from baseline, it is clear that there has been an improvement in the mean PTV readings for both the NS and S group with no statically significant differences between them. In the first 6 months from baseline, the change in mean PTV readings was slightly greater in the NS group than that in the S group, while after 12 months and 24 months, the change in PTV readings was greater in the S group than that in the NS group showing the greatest improvement at 12-month follow-up (−0.454 ± 1.6525, −1.775 ± 1.6637 (*p*=0.064)) (Table 4 and Figure 6).

## 5. Discussion

Implant stability is one of the important parameters that would influence the successful osseointegration of dental implants. Primary implant stability is the mechanical stability, whereas secondary implant stability is the biological phenomena and is the result of osseointegration [38]. Several methods have been used to measure primary and secondary implant stability, but Resonance Frequency Analysis (RFA) using Osstell and Periotest has been the most noninvasive method commonly used to objectively monitor implant stability at different observation periods [39–41]. In the present study, the Periotest was used to monitor the changes in secondary implant stability because the Osstell would require the smart peg to be attached to the implant, and so, that would require unscrewing of the attachment each time during measurement, so that was not applicable. Zix et al. have proved that the Periotest is more user friendly and time and cost efficient because the superstructure should not be removed when performing the measurements [42]. Despite the fact that both instruments are used to evaluate stability, Meredith et al. have reported that the Periotest has low reproducibility and sensitivity [43], while on the other hand, several studies concluded that the Periotest is a reliable method to objectively determine implant stability [15, 44–52]; furthermore, Khalaila et al. have concluded that the Periotest is a reliable tool for assessing implant stability and it would provide predictive information about marginal bone loss [53].

Interoperator and interinstrument variability have been considered to affect the Periotest scores such as the angulation and positioning of the device hand piece (horizontal distance and angle of the implant) [54]. In the following trial, the Periotest was held as a “pen grip” in the anterior area being perpendicular at the midbuccal area of the attachment as was described in the Periotest user manual by Schulte and Lukas [55] concluding that a single Periotest measurement will not allow prognosis for the stability of an implant, and so, that was the reason that 5 readings were recorded for each patient, and an average reading was then recorded in the patients file.

The bone quality and quantity are important factors that influence the primary implant stability. The more dense the bone, the better the initial stability [56]. In the present study, all implants were installed in the midline of the anterior mandible which is considered to be of dense bone as classified by Lekhom and Zarb [57], so all of the installed implants in both the submerged and nonsubmerged groups were of high initial stability with a mean PTV reading ranging from −4.4 to −5.8; Olive and Aparicio further confirmed that the PTV readings of dental implants lie between a narrow zone of −5 to +5, where −5 was considered to be high stability [44]. The initial stability will consequently influence secondary implant stability [58]; that is the reason why the PTV readings recorded in both groups, the submerged and nonsubmerged groups, after 3 months of healing were of high secondary stability, as the more negative values of the Periotest indicates greater implant stability [59]. Truhlar et al. concluded that the PTV at second-stage surgery is the best estimate for the Bone-Implant-Contact (BIC), as PTV determines implant stability and, more specifically, BIC which is mainly influenced by bone quality [51].

The results of the present trial are part of the randomized clinical trial that has randomized the patients at the day of pickup using sealed envelopes into two groups: ball and CM-LOC attachment. The results of the ball attachment was the scope of this clinical trial, mainly because the ball attachment has been used in several studies to retain the mandibular single implant retained overdenture and have achieved reliable outcomes [24, 31–34]. Very few studies have addressed the changes in stability for the NS and S groups using ball attachment, so that was the reason this clinical trial was conducted to add to the outcomes of the ball attachment used in a single implant retained overdenture.

There has been an improvement in PTV readings for the NS and the S group from the baseline (day of pickup) till 24-month follow-up without any significant difference between the groups. The PTV readings for the NS group was initially greater PTV at baseline (day of pickup) till 3-month follow-up when compared to the S group; then, starting from 6-month follow-up till 24-month follow-up, the S group has shown greater PTV when compared to NS. An explanation for this is that, during the 3-month healing period, the NS group had a healing abutment and the fitting surface of the denture was relieved by applying a soft liner to help reduce the forces falling on the installed implant for successful osseointegration. The NS group was subjected to more mechanical stimulation than the S group; this mechanical stimulation may have enhanced bone formation [60–62] in the NS than the S group. Branemark et al. have reported that new bone formed under loading conditions [63] consisted mainly of mature lamellar bone which is of greater density than the new bone formed under unloaded conditions; this phenomena has been referred to “form follows function” [64], so initially, the NS group had higher PTV readings than the S, while for the S group, the PTV readings have started to show greater scores after the pickup and loading of the attachments which have resulted in physiologic mechanical stimulation that consequently led to mature lamellar bone formation and, thus, greater bone to implant contact which consequently improved the PTV readings over 24-month follow-up. It is clear from the present study that, at 6-, 12-, and 24-month follow-up, the S has shown a greater improvement in the mean change in PTV readings than the NS as this would come in agreement with the work of Levy et al. where histomorphometric analysis revealed that bone-implant contact is greater for the submerged protocol [65].

## 6. Conclusions

Both the nonsubmerged and the submerged healing protocol have resulted in reliable Periotest readings using ball attachment for a single implant retained overdenture. The submerged group has resulted in a greater improvement change in Periotest readings after the 12- and 24-month follow-up period when compared to the nonsubmerged group although this improvement was not statistically significant.

### 6.1. Clinical Significance

The submerged healing protocol using ball attachment for a single implant retained mandibular overdenture would yield higher secondary stability than the nonsubmerged protocol after 24-month follow-up.

## Figures and Tables

**Table 1:** The distribution of patients throughout the follow-up intervals for all groups of patients.

	3 months after healing	Submerged		Nonsubmerged	
		Ball group	CM-LOC group	Ball group	CM-LOC group
Number of patients	71	15	18	19	19
Number of males	50	11	14	11	14
Number of females	21	4	4	8	5
Mean age of males (years)	60.4	61.8	59.3	59.1	61.7
Mean age of females (years)	60.8	64.75	58.5	63.1	57.2
Number of patients who refused Periotest reading		3 patients 2 males 1 female	3 patients All males	4 patients All males	5 patients 2 males 3 females
Number of dropouts at 3-month follow-up		1 patient died Male	—	—	1 patient Male
Number of dropouts at 6-month follow-up		2 patients 1 male 1 female	—	—	—
Number of dropouts at 9-month follow-up		1 patient died 1 male	—	—	1 patient died 1 male
Number of dropouts at 12-month follow-up		—	—	—	—
Number of dropouts at 24-month follow-up		—	2 patients All males	1 patient (hospitalized) Female	2 patients (hospitalized) All males

**Table 2:** The interobserver consistency using Cronbach's alpha statistics. NS: nonsubmerged and S: submerged.

	3 months after healing	3-month follow-up	6-month follow-up	9-month follow-up	12-month follow-up	24-month follow-up
Cronbach's alpha NS group	0.965	0.991	0.995	0.969	0.975	0.953
Cronbach's alpha S group	0.946	0.981	0.997	0.957	0.995	0.979

**Table 3:** The mean = M and standard deviation = SD for the nonsubmerged = NS and submerged = S groups of patients for the ball attachment group. *p* value ≤0.05 is considered statistically significant (^*∗*^).

		Baseline (at the day of pickup)	3-month follow-up	6-month follow-up	9-month follow-up	12-month follow-up	24-month follow-up
NS	M	−4.471	−4.84	−4.507	−4.808	−4.753	−5.730
	SD	1.489	1.326	1.3528	1.7689	1.7082	1.7804

S	M	−4.391	−4.67	−4.772	−5.314	−5.518	−5.855
	SD	1.4727	1.194	1.1256	1.1596	1.2521	1.2581

*p* value		0.913	0.732	0.801	0.451	0.246	1

**Table 4:** The mean = M and standard deviation = SD for the nonsubmerged = NS and submerged = S groups of patients for the ball attachment group. *p* value ≤0.05 is considered statistically significant (^*∗*^).

		Baseline 6-month follow-up	Baseline 12-month follow-up	Baseline to 24-month follow-up
NS	M	−0.346	−0.454	−1.412
	SD	1.1927	1.6525	1.9129

S	M	−0.333	−1.775	−1.963
	SD	1.9947	1.6637	2.4278

*p* value		0.650	0.064	0.374

## Data Availability

A protocol for the randomized clinical study is available from the corresponding author and would be sent when required.

## Abbreviations

PTV: Periotest damping effect
CM-LOC: Cendres and Metaux Locator
NS: Nonsubmerged
S group: Submerged.
